# Epidemiological Risk Analysis of Home Injuries in Italy (1999–2006)

**DOI:** 10.3390/ijerph110404402

**Published:** 2014-04-22

**Authors:** Pierpaolo Ferrante, Alessandro Marinaccio, Sergio Iavicoli

**Affiliations:** Occupational Medicine Department (DML), National Workers Compensation Authority (INAIL), Research Area, Via Stefano Gradi 55, Rome 00143, Italy; E-Mails: a.marinaccio@inail.it (A.M.); s.iavicoli@inail.it (S.I.)

**Keywords:** home injuries, Poisson regression, risk analysis

## Abstract

Home injuries are an important public health issue in both developed and developing countries. This study focused on the Italian epidemiological framework between 1999 and 2006, using a nation-representative sample provided by the National Institute of Statistics. Every year, about 3,000,000 Italian residents reported at least one home injury, with an overall annual rate of 5.2/100 (95% CI 5.1–5.4); 3.2/100 (3.0–3.4) for males and 7.2/100 (6.9–7.4) for females. Poisson regression models were used for different age-specific populations (children, young/adults and older people), to evaluate the effects of socio-demographic, health/income satisfaction and housing variables. For children, non-applicable variables (including smoking and health satisfaction) were taken as those of the head of family, while housework time was taken the family mean time. Evidence of decreasing time trend in risk of home injury was found only among young/adults (*p* < 0.01). The following were risk factors: female gender (adjusted relative risk—RR 2.0 for older people and RR 1.9 for young/adults, *p* < 0.01); one additional hour of work at home (RR 1.009, *p* < 0.01 for young/adults and RR 1.016, *p* = 0.01 for children); smoking (RR 1.3, *p* < 0.01 for young/adults and *p* = 0.02 for children); health dissatisfaction (RR 1.3, *p* = 0.05 for children, RR 1.6 for young/adults and RR 1.7 for older people, *p* < 0.01); income dissatisfaction (RR 1.2, *p* < 0.01 for young/adults ); living alone (RR 1.5, *p* < 0.01 for young/adults and RR 1.2, *p* < 0.02 for the older people); having a garden (RR 1.1, *p* < 0.01 for young/adults ). Awareness of the need for safety at home could be boosted by information campaigns on the risk, and its social cost could be reduced by specific prevention schemes. Developing tools for assessing the risk at home and for removing the main hazards would be useful for both informative and prevention interventions.

## 1. Introduction

Home injuries are a significant public health problem in both developed and developing countries. In the USA during the 1997–2007 period most injuries occurred at home and more than 40% of reported medically treated injuries occurred in and around the home [1,2]. In England and Wales there were about 3,500 deaths from injury in and around the home in 2005, accounting for 70% of non-transportation fatalities [3]. Between 2003–2005, the overall annual rate of fatal home and leisure accidents in the 27 European Union Member States was 22 per 100,000 inhabitants. This is more than ten times the rate of fatal workplace accidents (1.5 per 100,000) and more than twice the rate of fatal road traffic accidents (10 per 100,000) [4].

Children aged 0–14 years and the older people aged over 70 spend a lot of time at home, so they are more exposed to the risks of home injury. Their injuries need the most specialized health care and, in countries with a welfare and public health system (such as Italy), this implies high social costs. An estimated 6% of Australian children require emergency department treatment for a new injury each year and 15% of presentations lead to hospital admission [5,6]. In New Zealand, a childhood cohort study estimated the percentages of injury requiring medical treatment as 11% at ages 6 and 7, 19% at ages 10 and 11, and 25% in 14- and 15-year-olds [7,8]. Annually in England and Wales, more than 300,000 people older than 65 years require emergency department assistance because of home injuries, falls being the most important [9].

In Italy, housing health and safety were regulated by Law No. 493/1999 (3 December 1999); it established institutional roles and marked the starting point for national coordinated actions concerning home injuries [10]. The Ministry of Health plan policies, the National Institute of Health (ISS) and the Italian Institute of Statistics (ISTAT) provide figures on the topic. ISS set up the National Information System about Home Injury (SINIACA), which is linked to the European Injury Database [11]. SINIACA collects hospital admissions, emergency department attendances and fatal events (as recorded on death certificates) for home injuries; it does not consider self-treated injuries or those dealt with by a pharmacy, outpatient department or physician at home. ISTAT runs an annual multipurpose survey section called “Daily Life” (DL), which covers all non-fatal events occurred at home due to a home injury cause (Yes, No). At irregular times (the last one dates from 1999), DL also contained injury details such as room of occurrence, injury consequence and received assistance.

Several studies have analyzed the SINIACA data on a regional level. Farchi *et al.* [12] tested the data quality by comparing different operational definitions of home accident mortality. Panatto *et al.* [13] investigated injury data for older people (aged 65–92 years) collected by three hospitals in Genoa, while Majori *et al.* [14] analyzed injuries reported by one hospital in Verona. The DL survey was analysed by Snidero *et al.* [15] and Ferrante *et al*. [16]. The former focused on the geographical distribution of domestic injuries, the latter on the patterns of injury and the most exposed people.

So far, Italian studies have focused on specific features and they do not provide a broad overview to the public health authorities for planning interventions. With a view to suggesting opportunities for efficient prevention, this study investigated multiple aspects of the phenomenon such as magnitude, time trends after the current law about prevention ([10]) was passed (3 December 1999), effects of socio-demographic, health/income satisfaction and housing variables on injury risks over a broad period (1999–2006) for three different populations: children (0–14 years), young/adults (15–64 years) and older people (≥65 years). Injuries at home are largely preventable, and risks analysis findings could support both information and prevention public health interventions. Awareness of the need for safety at home could be boosted by information campaigns on the risk, and the social cost could be reduced by specific prevention schemes.

## 2. Methods

The DL section of the ISTAT Italian multipurpose survey was the data source (1999–2006). For each family member, the survey was based on face-to-face interviews and a self-administrated questionnaire; when needed children were supported by guardians, information regarding unavailable persons were provided by a relative (proxy interviews). The two-stage sample covers 141,583 families and 374,471 interviews (approximately equally distributed among years) and is representative of the whole nation. The response rate was over 95%, the percentage of proxy interviews was 25.6%, 61.4% among children (0–14 years), 21.2% among young/adults (15–64 years) and 13.3% among older people (≥ 65 years). In the first stage, municipalities were divided into two groups: self-representative municipalities (SR), with larger populations, and non-self-representative municipalities (NSR), *i.e.*, those remaining. For discriminating among SR and NSR, the population cutoff was determined at regional level by the populations’ ratio region/Italy and the mean number of families’ components. In the SR, each municipality was considered an independent layer and cluster sampling was applied. The primary sampling units comprised households entered in the Public Registry. In the NSR, a two-stage pattern was adopted: municipalities themselves and the households in the Public Registry were respectively the primary and secondary units. Both units were sampled without replacement, the primary ones with probabilities proportional to their population size and the secondary ones by a uniform distribution; sample details can be found elsewhere [17,18,19,20,21,22,23].

**Figure 1:**
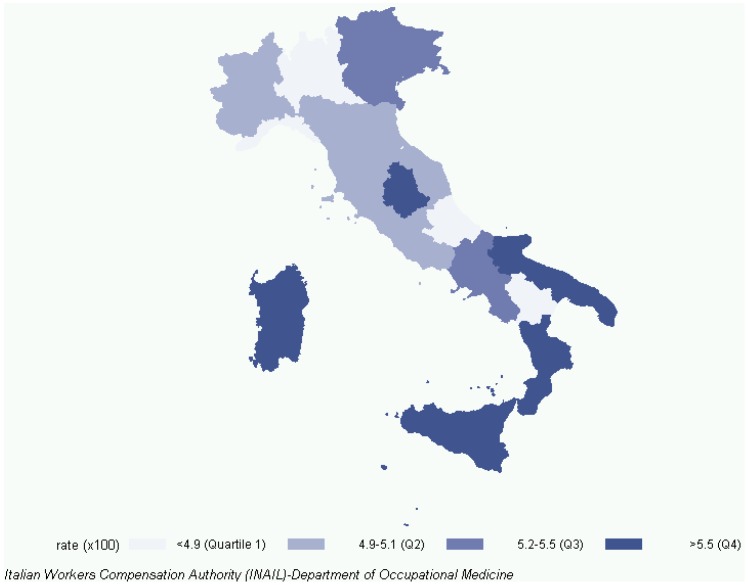
Age-adjusted rates of home injury in Italy (1999–2006) by region (per 100 inhabitants).

The home injury item was: In the last three months, have you experienced at least one accident at home with injuries? As the recorded events referred to a quarterly value, the annual number (AN) and the annual rate (AR) per 100 inhabitants of home injured were calculated by multiplying the occurrences by four. Confidence intervals (95%) for AR were computed by sample properties. Regional age-adjusted rates were assessed and presented as map (Figure 1). Three multivariate analyses were done, by Poisson regression, referring to children (aged 0–14 years), young/adults (aged 15–64 years) and the older people (aged ≥ 65 years). To account for the hierarchical nature of the data (family members), family was the grouping variable of the generalized estimating equations method (repeated results of the SAS GENMOD procedure).

The explanatory variables were the same for all the models: year (1999, 2000, 2001, 2002, 2003, 2005, 2006), macro-region (North East, North West, Center, South and the islands), sex (male or female), age (11 levels: 0–5, 6–14, 15–24, 25–34, 35–44, 45–54, 55–64, 65–69, 70–74, 75–79, ≥80 years), marital status (single, married, divorced, widowed), educational level (elementary, middle school, high school, university degree), employment status (housewife, employed, unemployed, student, retired, other), number of people in family (1, 2, >2), housework (weekly hours), number of rooms (1–3, >3), home ownership (yes, no), smoker (current, ex, never), income dissatisfaction (yes, no) and health dissatisfaction (yes, no). Reference modalities were those with the lowest values detected. Finally, interaction between sex and age was included. As children’s safety depends on their parents, for the non-applicable variables marital status, education, employment status, smoking, income/health dissatisfaction and home ownership were considered those of the Head of Family (HoF) while Housework weekly time was taken the mean of the family. The HoF was indicated by the family as the reference member. With respect to the child, he/she was generally the father (84.6%), sometimes the mother (10.8%), rarely the grandfather (2.9%) or the grandmother (1.3%) or anyone else (0.4%).

Relative risks adjusted for all independent variables included in the Poisson model (*i.e.*, socio-demographic, income/health satisfaction and housing variables) are presented in Table 3. The linear time trend was tested by considering year as a continuous variable. We used SAS package, version 9.3 (SAS Institute Inc., Cary, NC, USA), for statistical analysis.

## 3. Results

In Italy more than three million persons per year had at least one home injury between 1999 and 2006, with an overall crude annual incidence rate of 5.2/100; 3.2/100 for males and 7.2/100 for females. Female rate (AR 7.2) was twice the male one (AR 3.2). The age-class distribution of risk rates differed by sex: babies (0–5 years) and people over 80 years were the groups most at risk among respectively the male and female populations (Table 1).

**Table 1:** Annual number and annual rate per 100 inhabitants with corresponding 95% confidence interval by year ^a^ and age group for males and females.

	Male			Female			Total		
**Variable**	***AN***	***ARx100***	**95% CI**	***AN***	***ARx100***	**95% CI**	***AN***	***ARx100***	**95% CI**
Year									
1999	693286	2.5	(2.0–3.0)	2335703	8.0	(7.2–8.7)	3028989	5.3	(4.9–5.7)
2000	907376	3.3	(2.7–3.8)	2375648	8.1	(7.3–8.8)	3283024	5.7	(5.3–6.2)
2001	846003	3.0	(2.5–3.6)	2001186	6.8	(6.1–7.5)	2847189	5.0	(4.5–5.4)
2002	908929	3.3	(2.7–3.8)	1940374	6.6	(5.9–7.3)	2849303	5.0	(4.5–5.4)
2003	868492	3.1	(2.6–3.6)	1943385	6.6	(5.9–7.3)	2811877	4.9	(4.5–5.3)
2005	873679	3.1	(2.6–3.6)	2168591	7.3	(6.5–8.1)	3042270	5.2	(4.8–5.7)
2006	1147014	4.0	(3.4–4.7)	2042462	6.8	(6.1–7.6)	3189476	5.5	(5.0–5.9)
Age									
0–5	873560	7.5	(6.0–9.0)	528540	4.8	(3.6–6.0)	1402100	6.2	(5.3–7.1)
6–14	545746	3.0	(2.2–3.7)	424683	2.5	(1.8–3.2)	970429	2.7	(2.2–3.2)
15–24	444209	2.0	(1.4–2.5)	765041	3.5	(2.8–4.2)	1209250	2.7	(2.3–3.1)
25–34	633368	2.1	(1.6–2.6)	1775302	6.1	(5.3–6.9)	2408670	4.1	(3.6–4.5)
35–44	872753	2.7	(2.1–3.2)	2216823	6.8	(6.0–7.6)	3089576	4.7	(4.3–5.2)
45–54	713875	2.7	(2.1–3.3)	2428447	8.9	(7.9–9.9)	3142322	5.9	(5.3–6.4)
55–64	824565	3.5	(2.8–4.2)	1989438	8.2	(7.2–9.2)	2814003	5.9	(5.3–6.5)
65–69	350474	3.5	(2.4–4.5)	1089575	9.1	(7.5–10.6)	1440049	6.5	(5.5–7.4)
70–74	330865	3.8	(2.5–5.0)	1071757	9.5	(7.9–11.2)	1402622	7.0	(6.0–8.0)
75–79	289419	4.6	(3.0–6.1)	1002622	10.9	(9.0–12.8)	1292041	8.3	(7.0–9.6)
>80	365945	6.7	(4.6–8.7)	1515121	14.0	(12.0–16.0)	1881066	11.5	(10.0–13.0)
Annual mean	892771	3.2	(3.0–3.4)	2114998	7.2	(6.9–7.4)	3007769	5.2	(5.1–5.4)

^a^ In 2004 no survey was done.

Throughout the whole period, age-adjusted injuries incidence rates were highest in the Islands, South and North East, while the North West and center had the lowest rates (Figure 1). For the three populations (children, young/adults and older people), simple associations between home injuries and the selected variables are shown in Table 2. Macro-region, sex and education were risk factors for all three populations. Effects of marital status, employment, number of family members, smoking, income and health dissatisfaction appeared significant only for young/adults and older people. For the three populations, Table 3 shows relative risks adjusted for socio-demographic, income/health satisfaction and housing variables, provided by the Poisson regression. For young/adults, risk in 1999 was significant higher (*p* = 0.01) than the reference year (2005) and evidence of decreasing time trend in risk of injuries was found (*p* < 0.001). Home safety was best in the North Western regions; the risks of injury for children and older people were highest in the North East (NE), and for young/adults in the Southern and central regions and the Islands. Married individuals were more exposed to risk of injury than single young/adults (RR 1.7, *p* < 0.01), divorced/separated (RR 1.3, *p* = 0.01) and widowed people (RR 1.4, *p* = 0.01); housewives (RR 1.2, *p* = 0.01) and the unemployed (RR 1.3, *p* < 0.01) were more exposed to risk of injury than employed people. Children with a student HoF had four times the risk of those with an employed HoF (RR 4.2, *p* = 0.04). Living alone is associated with an increased risk only for young/adults (RR 1.5, *p* < 0.01), but after dropping marital status from the model its effect also appeared as significant among the older people (remaining RR estimates and *p*-values of the reduced model (used only for older people) did not change in a significant way; RR 1.2, *p* = 0.02). A garden increased the risk of injury by the same magnitude for all three populations (RR 1.1), but its effect was significant only for young/adults (*p* < 0.01). The number of rooms, home ownership and the presence/absence of a balcony did not change the risk. Young/adults current and ex-smokers had a greater risk of injury (respectively RR 1.3 and RR 1.2, *p* < 0.01) than non-smokers; children living with smokers were also more strongly exposed to the risk of injury (RR 1.3, *p* = 0.02).

Health dissatisfaction (of HoF for children) increased the probability of injury (RR 1.3, *p* = 0.05 for children; RR 1.6, *p* < 0.01 for young/adults; RR 1.7, *p* < 0.01 for the older people) as did income dissatisfaction for young/adults (RR 1.2, *p* < 0.01). Each weekly hour of work at home increased the relative risk by 16% for children (*p* = 0.01) and by 9% for young/adults (*p* < 0.01). Sex differences are summarized in Table 4: males were most vulnerable at age ≤ 14 years and females in the over-14 groups.

## 4. Discussion

With an observed annual incidence rate of about 5 per 100 inhabitants, unintentional injuries at home are a significant source of morbidity, as well as a major societal cost burden [24,25,26,27]. The present analyses were based on representative nation-wide data about all non-fatal home injuries, recorded in the Italian multipurpose survey on daily life in the period 1999–2006. Age-adjusted risk rate distribution by regions and the adjusted relative risks by socio-demographic, income/health satisfaction and housing variables were assessed.

A preliminary analysis of limitations of the study is necessary. Details of the types of injury were not available in the data collections. Fatal injuries are not considered because of the nature of the survey (face-to-face interviews and a self-administered questionnaire). Surveys contained a non-negligible percentage of proxy responses (25.6%). The annual estimates of occurrence and rates did not consider seasonal patterns: collected injuries were relative to the first/last quarters of the year, and in the summer the scenario might be different. Finally, income and health were assessed by perceived measures and not through observed data.

Except for the North East, there was a territorial gradient, with home safety increasing from south to north (Figure 1). As noted by Snidero [15], the overall rate in the North-East is close to that of the South and Islands because of the high risk among children and older people (Table 3). In north-eastern regions, the highest injury rates among children and older people were accompanied by the highest alcohol consumption [28]; as the association between child and elder maltreatment with alcohol use is well known [29,30], maybe some episode of domestic violence could be recorded as home injury. This issue should be better investigated. The smallest percentage of people lunching at home was observed in northern regions [31], presumably associated with smallest propensity to stay at home, *i.e.*, reducing exposure time.

**Table 2:** Numbers and percentages of people injured according to selected variables, with corresponding *p*-values.

Variables	Children (0–14 years) ^a,b^					Young/adults (15–64 years)					Older people (65+ years)				
	Uninjured		Injured			Uninjured		Injured			Uninjured		Injured		
	n	(%)	n	(%)	*p*	n	(%)	n	(%)	*p*	n	(%)	n	(%)	*p*
**Year**					0.40					0.09					0.19
1999	8267	(99.1)	77	(0.9)		37,744	(98.7)	493	(1.3)		8825	(98.1)	175	(1.9)	
2000	8541	(98.8)	104	(1.2)		39,497	(98.7)	502	(1.3)		9782	(97.7)	227	(2.3)	
2001	7645	(99.0)	78	(1.0)		35,485	(98.9)	390	(1.1)		9290	(98.0)	192	(2.0)	
2002	7860	(99.0)	82	(1.0)		36,905	(98.8)	433	(1.2)		9863	(98.2)	184	(1.8)	
2003	7529	(99.2)	64	(0.8)		35,901	(98.8)	432	(1.2)		9594	(98.1)	188	(1.9)	
2005	7083	(99.0)	69	(1.0)		32,599	(98.9)	368	(1.1)		9645	(97.7)	224	(2.3)	
2006	6755	(99.0)	70	(1.0)		31,781	(98.9)	359	(1.1)		8989	(98.0)	180	(2.0)	
**Macro-region**					<0.01					<0.01					0.01
North west	9476	(99.0)	94	(1.0)		51,307	(99.0)	512	(1.0)		14,324	(98.3)	248	(1.7)	
North east	10,974	(98.7)	145	(1.3)		52,080	(98.9)	594	(1.1)		13,828	(97.8)	314	(2.2)	
Centre	8977	(99.0)	94	(1.0)		45,664	(98.8)	537	(1.2)		13,775	(98.0)	277	(2.0)	
South	17,698	(99.2)	149	(0.8)		73,168	(98.8)	911	(1.2)		17,895	(97.8)	395	(2.2)	
Major Islands	6555	(99.1)	62	(0.9)		27,693	(98.5)	423	(1.5)		6166	(97.8)	136	(2.2)	
**Sex**					<0.01					<0.01					<0.01
Male	27,752	(98.8)	328	(1.2)		124,241	(99.3)	831	(0.7)		28,427	(98.9)	322	(1.1)	
Female	25,928	(99.2)	216	(0.8)		125,671	(98.3)	2146	(1.7)		37,561	(97.3)	1048	(2.7)	
**Marital Status**					0.67					<0.01					<0.01
Single	1475	(98.9)	16	(1.1)		89,157	(99.3)	622	(0.7)		4558	(98.2)	85	(1.8)	
Married	47,653	(99.0)	486	(1.0)		142,602	(98.6)	2085	(1.4)		38,223	(98.4)	604	(1.6)	
Divorce	2992	(99.0)	31	(1.0)		12,185	(98.7)	159	(1.3)		1478	(97.8)	34	(2.2)	
Widowed	1560	(99.3)	11	(0.7)		5968	(98.2)	111	(1.8)		21,729	(97.1)	647	(2.9)	
**Education**					0.01					<0.01					<0.01
Elementary	6116	(99.0)	62	(1.0)		41,179	(98.4)	658	(1.6)		49,726	(97.8)	1101	(2.2)	
Middle school	21,770	(99.2)	186	(0.9)		91,795	(98.9)	1041	(1.1)		8057	(98.4)	127	(1.6)	
High school	20,083	(98.9)	221	(1.1)		94,450	(98.9)	1035	(1.1)		6068	(98.2)	109	(1.8)	
Degree/PhD	5708	(98.7)	75	(1.3)		22,488	(98.9)	243	(1.1)		2137	(98.5)	33	(1.5)	
Missing	3	(100)	0	(0.0)		0	(0.0)	0	(0.0)		0	(0.0)	0	(0.0)	
**Employment status**					0.15					<0.01					<0.01
Employed	46,667	(99.0)	473	(1.0)		138,058	(99.0)	1330	(1.0)		1718	(99.1)	15	(0.9)	
Student	38	(95.0)	2	(5.0)		28,153	(99.5)	155	(0.5)		-	-	-	-	
Housewife	1525	(99.1)	14	(0.9)		37,339	(97.7)	898	(2.3)		11,322	(97.4)	300	(2.6)	
Retired	2196	(99.0)	22	(1.0)		21,120	(98.7)	278	(1.3)		46204	(98.2)	845	(1.8)	
Unemployed	2366	(99.1)	21	(0.9)		18,417	(98.8)	224	(1.2)		-	-	-	-	
Other	873	(98.6)	12	(1.4)		6825	(98.7)	92	(1.3)		6698	(97.0)	210	(3.0)	
Missing	15	(100)	0	(0.0)		0	(0.0)	0	(0.0)		46	(100)	0	(0.0)	
**No. family members**					0.41					<0.01					<0.01
1	-	-	-	-		15,478	98.7	198	1.3		17,230	97.3	485	2.7	
2	1200	98.8	15	1.2		40,691	98.7	547	1.3		30,883	98.2	560	1.8	
>2	52,480	99.0	529	1.0		193,743	98.9	2232	1.1		17,875	98.2	325	1.8	
**Housework (weekly hours)**					0.01					<0.01					<0.01
mean (sd)	11.6	(6.5)	12.5	(6.7)		17.2	(20.1)	28.3	(23.4)		19.0	(19.2)	21.9+	(20.7)	
**Smoking**					0.20					0.01					<0.01
Current	18,365	(98.9)	204	(1.1)		66,784	(98.7)	847	(1.3)		6536	(98.6)	91	(1.4)	
Ex	14,223	(98.9)	151	(1.1)		47,641	(98.8)	602	(1.3)		19,069	(98.4)	316	(1.6)	
Never	20,204	(99.1)	188	(0.9)		129,664	(98.9)	1469	(1.1)		39,070	(97.7)	939	(2.3)	
Missing	888	(99.9)	1	(0.1)		5823	(99.0)	59	(1.0)		1313	(98.2)	24	(1.8)	
**Income dissatisfaction**					0.42					<0.01					<0.01
Yes	22,360	(99.0)	221	(1.0)		100,150	(98.6)	1397	(1.4)		28,147	(97.7)	659	(2.3)	
No	30,244	(98.9)	321	(1.1)		142,871	(99.0)	1510	(1.0)		36,133	(98.2)	679	(1.8)	
Missing	1076	(99.8)	2	(0.2)		6891	(99.0)	70	(1.0)		1708	(98.2)	32	(1.8)	
**Health dissatisfaction**					0.14					<0.01					<0.01
Yes	4947	(98.8)	61	(1.2)		28,090	(97.8)	619	(2.2)		26,849	(97.2)	779	(2.8)	
No	47,613	(99.0)	480	(1.0)		214,914	(98.9)	2286	(1.1)		37,454	(98.5)	559	(1.5)	
Missing	1120	(99.7)	3	(0.3)		6908	(99.0)	72	(1.0)		1685	(98.1)	32	(1.9)	
**Home owner**					0.36					<0.01					0.07
No	17,282	(98.9)	185	(1.1)		61,417	(98.7)	800	(1.3)		13,136	(97.8)	301	(2.2)	
Yes	35,522	(99.0)	350	(1.0)		184,603	(98.9)	2124	(1.1)		51,800	(98.0)	1050	(2.0)	
Missing	876	(99.0)	9	(1.0)		3892	(98.7)	53	(1.3)		1052	(98.2)	19	(1.8)	
**No. of rooms**					0.45					0.01					0.30
1–3	11,137	(98.9)	120	(1.1)		53,167	(98.7)	690	(1.3)		20,809	(97.9)	450	(2.1)	
>3	42,543	(99.0)	424	(1.0)		196,745	(98.9)	2287	(1.1)		45,179	(98.0)	920	(2.0)	
**At least one balcony**					0.42					0.69					0.62
No	8475	(99.1)	79	(0.9)		41,638	(98.8)	502	(1.2)		15,758	(97.9)	336	(2.1)	
Yes	44,433	(99.0)	457	(1.0)		204,873	(98.8)	2421	(1.2)		49,278	(98.0)	1018	(2.0)	
Missing	772	(99.0)	8	(1.0)		3401	(98.4)	54	(1.6)		952	(98.3)	16	(1.7)	
**Home with garden**					0.11					0.17					0.51
No	29,280	(99.0)	281	(1.0)		134,937	(98.8)	1576	(1.2)		38,077	(98.0)	780	(2.0)	
Yes	21,965	(98.9)	243	(1.1)		104,520	(98.8)	1287	(1.2)		25,016	(97.9)	532	(2.1)	
Missing	2435	(99.2)	20	(0.8)		10,455	(98.9)	114	(1.1)		2895	(98.0)	58	(2.0)	

^a^ Marital status, Education, Employment status, Smoking, Income/ Health dissatisfaction and Home ownership refer to the family head. ^b^ Hours of housework refer to the mean of the family.

**Table 3:** Adjusted risk ratios with correspondent *p*-values.

	Children (0–14 years) ^a,b^		Young/adults (15–64 years)		Older people (65+ years)	
Factor	RR	*p*	RR	*p*	RR	*p*
**Year**						
1999	1.1	0.48	1.2	0.01	1.1	0.46
2000	1.3	0.08	1.1	0.23	1.2	0.06
2001	1.2	0.41	1.0	0.53	1.1	0.27
2002	1.2	0.23	1.0	1.00	1.0	1.00
2003	1.0	0.81	1.0	0.77	1.0	0.98
2006	1.2	0.45	0.9	0.23	1.2	0.11
2005	1.0	-	1.0	-	1.0	-
**Macro-region**						
North East	1.3	0.04	1.1	0.10	1.3	0.01
Centre	1.1	0.66	1.2	0.02	1.1	0.40
South	0.9	0.33	1.2	<0.01	1.2	0.04
Major Islands	1.1	0.77	1.4	<0.01	1.2	0.10
North west	1.0	-	1.0	-	1.0	-
Sex						
Female	0.8	<0.01	1.9	<0.01	2.0	<0.01
Male	1.0	-	1.0	-	1.0	-
**Marital status**						
Divorced	1.3	0.49	1.3	0.01	1.5	0.06
Married	1.3	0.31	1.7	<0.01	1.0	0.74
Widowed	0.9	0.72	1.4	0.01	1.3	0.06
Single	1.0	-	1.0	-	1.0	-
**Education**						
Degree/PhD	1.4	0.11	1.1	0.27	0.9	0.49
High school	1.0	0.89	1.1	0.06	1.1	0.64
Middle school	0.8	0.13	1.0	0.43	0.9	0.17
Elementary	1.0	-	1.0	-	1.0	-
**Employment status**						
Housewife	1.0	0.93	1.2	0.01	1.4	0.24
Other	1.3	0.37	1.2	0.20	1.7	0.07
Retired	1.0	0.92	1.0	0.96	1.4	0.25
Student	4.2	0.04	0.9	0.33	-	-
Unemployed	0.9	0.78	1.3	<0.01	-	-
Employed	1.0	-	1.0	-	1.0	-
**No. family members**						
1	-	-	1.5	<0.01	1.1 ^c^	0.22 ^c^
2	1.3	0.39	1.1	0.05	1.0	0.72
>2	1.0	-	1.0	-	1.0	-
Housework (weekly hours)	1.016	0.01	1.009	<0.01	1.003	0.06
**Smoking**						
Current	1.3	0.02	1.3	<0.01	1.0	0.77
Ex	1.2	0.11	1.2	<0.01	1.2	0.07
Never	1.0	-	1.0	-	1.0	-
**Income dissatisfaction**						
Yes	1.0	0.87	1.2	<0.01	1.0	0.92
No	1.0	-	1.0	-	1.0	-
**Health dissatisfaction**						
Yes	1.3	0.05	1.6	<0.01	1.7	<0.01
No	1.0	-	1.0	-	1.0	-
**Home owner**						
No	1.1	0.38	1.1	0.08	1.0	0.52
Yes	1.0	-	1.0	-	1.0	-
**Number of rooms**						
>3	0.9	0.45	0.9	0.28	1.0	0.78
1–3	1.0	-	1.0	-	1.0	-
**At least one balcony**						
Yes	1.2	0.12	1.1	0.30	1.1	0.25
No	1.0	-	1.0	-	1.0	-
**Home with garden**						
Yes	1.1	0.2	1.1	<0.01	1.1	0.08
No	1.0	-	1.0	-	1.0	-

Notes: ^a^ Marital status, Education, Employment status, Hours worked at home, Smoking, Income/Health dissatisfaction and Home ownership refer to the family head. ^b^ Hours of housework refer to the mean of the family. ^c^ Without marital status variable, the Poisson model estimated RR = 1.2 with *p* = 0.02; the remaining RR estimates and *p*-values did not change in a significant way.

**Table 4:** Adjusted risk ratios for males and females by age, with corresponding *p*-values.

Age (years)	Males *vs.* Females	
	RR	*p*
0–5	1.4	<0.01
6–14	1.3	0.09
15–24	0.6	<0.01
25–34	0.5	<0.01
35–44	0.5	<0.01
45–54	0.5	<0.01
55–64	0.6	<0.01
65–69	0.5	<0.01
70–74	0.4	<0.01
75–79	0.5	<0.01
>80	0.6	<0.01

Analysis by year provides information about the effects of the current law from its establishment (3 December 1999) up to seven years later. Poisson regression results showed a high risk in 1999 for young/adults and evidenced a decreasing time trend among them; as the time effect was adjusted for all the other independent variables of the model, this finding suggests the need to improve age-specific policies for children and older people. 

Age patterns of rates differed for the sexes, indicating different probabilities for types and consequences of injury: males are exposed mainly to cuts and bumps while females are prone to burns and falls [16]. Living alone increased the risk for young/adults and older people, students’ HoF (presumably young and then inexperienced) strongly raised the risk for children; as expected, persons spending a lot of time at home were the most exposed to risk, such as young/adults unemployed, housewives and widowed people. Each added weekly hour of work at home (of the HoF for children) increased the relative risk of injury by 16‰ for children, 3‰ for older people and 9‰ for young/adults. 

These results highlight the importance of information campaigns [32], above all for young parents, and of promoting guiding actions to remove the main home hazards [33,34]; implementing tools for assessing injury risks at home could support both information and prevention public health policies.

Simple interventions for children and parents could be done by schools, where validated scales of risks could be distributed. Annual assessments of injury risks at home could be assigned to the students and decreasing trends among years could be rewarded. Housewives and older people could be reached in their homes by television. Advice for improving safety at home could be provided in popular entertainment programs and, as for traffic accidents, annual national statistics could be divulged by news programs in prime time. Places of worship could be effective as a promoting communication channel as most exposed people (old women, above all in the South/Islands) go there very regularly [31]. Specific interventions provided as face-to-face education, especially with the provision of safety equipment, could be used for increasing safety practices [35].

Supporting the relation between household income and home injuries [36,37], income dissatisfaction was a significant risk factor for young/adults and is presumably associated with low income. Health dissatisfaction increased the risk of injury at home among young/adults and the older people. The underlying structure of associations among diseases, drugs and home injury risks could be the content for future researches. 

Among the housing variables, attention needs to focus on the garden where the risk of injury was significantly high only for young/adults; specific garden hazard studies have been done, and measures for prevention suggested [38]. As men are specially exposed during repairs [16], large distribution chains of gardening and do-it-yourself equipment (often unified) could be boosted to promote the right working techniques. The number of rooms, home ownership and presence/absence of balconies did not change the injury risk. Young/adults ex- and current smokers and children living with smokers had a greater risk of injury than the non-smokers, presumably due to differences in lifestyle.

To follow the progress of these home risks and integrate them with the injury patterns [16], the survey section with injury details needs to be repeated at least every five years. In addition, to permit national and European comparisons, injury items need to conform to those used by the SINIACA. To remove the main hazards in the home, user-friendly tools to assess the safety at home, such as the “home risk indicator” [39], should be validated and implemented.

## 5. Conclusions

What is already known on this topic:

- Children and older people are the most exposed people.
- Risk distribution among ages are different by gender.

What this study adds:

- We estimate the magnitude and trend of home injuries in Italy in an broad period (1999–2006) starting just after the establishment of the current law (No. 493/1999) about home injury.
- We examine injury risks for different age-specific populations (children, young/adults and older people) and assess the effects of socio-demographic, health/income satisfaction and housing variables.
- We find a decreasing trend of risk only in young/adults suggesting specific information campaigns.
